# Towards Sustainable Carbon Return from Waste to Industry via C_2_-Type Molecular Unit

**DOI:** 10.3390/ijms231911828

**Published:** 2022-10-05

**Authors:** Konstantin S. Rodygin, Kristina A. Lotsman, Dmitriy E. Samoylenko, Viktor M. Kuznetsov, Valentine P. Ananikov

**Affiliations:** 1 Institute of Chemistry, Saint Petersburg State University, Universitetskiy pr. 26, 198504 Saint Petersburg, Russia; 2 N. D. Zelinsky Institute of Organic Chemistry, Russian Academy of Sciences, Leninsky pr. 47, 119991 Moscow, Russia

**Keywords:** calcium carbide, acetylene, pyrolysis, municipal waste

## Abstract

A general possibility of a sustainable cycle for carbon return to high-value-added products is discussed by turning wastes into acetylene. Pyrolyzed solid municipal wastes, pyrolyzed used cationic exchangers, and other waste carbon sources were studied in view of the design of a sustainable cycle for producing calcium carbide and acetylene. The yields of calcium carbide from carbon wastes were as high as those from industrial fossil raw materials (coke, charcoal, etc.). Conversion of carbon-containing wastes to calcium carbide provides an excellent opportunity to make acetylene, which is directly compatible with modern industry. Overall, the process returns carbon-containing wastes back to sustainable cycles to produce high-value-added products involving only C_2_-type molecules (calcium carbide and acetylene). Calcium carbide may be stored and transported, and on-demand acetylene generation is easy to realize. Upon incorporation into the waste processing route, calcium carbide may be an efficient carbon reservoir for quick industrial uptake.

## 1. Introduction

Waste recycling involving thermochemical treatment is a universal approach and an important component of a circular economy [1,2,3,4]. Pyrolysis is an efficient way of waste recycling, allowing the recovery of valuable feedstock from waste [5,6]. The key advantage of pyrolysis [7] and thermochemical methods [8] is their versatility and applicability to various types of processed materials, including mixtures of these materials. For example, a wide variety of plastics can be successfully processed individually or in mixtures. The importance of this area recalls urgent strategies for further usage of pyrolyzed products. Indeed, considering the amount of waste processed, a massive amount of carbon can be potentially produced by pyrolysis [9,10].

Efficient use of carbon is one of the major challenges in the circular economy [11,12,13,14,15,16]. The end-of-life of used materials is accompanied by certain carbon losses in the form of waste. Conventional incineration [17] of waste results in carbon dioxide emissions (approximately 1 ton of CO_2_ per 1 ton of waste) ruining the carbon neutral cycle.

**Figure 1:**
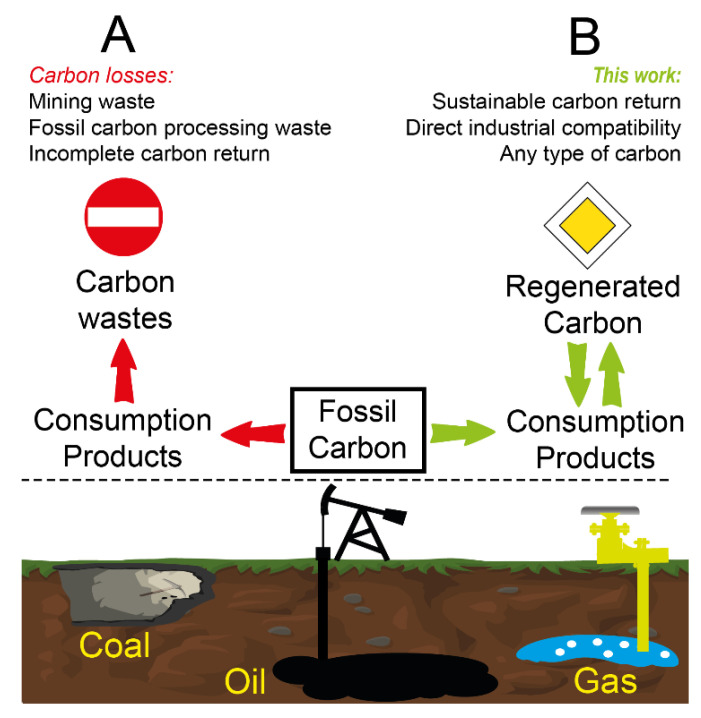
Carbon losses in the current route of fossil utilization (**A**, red arrows) and possible improvement via the regeneration of carbon described in this article (**B**, green arrows).

In fact, mined fossil carbon is transformed into consumption products followed by unpreventable carbon wastes (Figure 1A). Of course, many options are now considered for developing approaches that can be used to move towards the circular economy [18,19,20,21,22,23,24]. Regeneration of carbon after using consumption products should return carbon, decreasing disposal areas and reducing the required amount of fossil carbon (Figure 1B). Pyrolysis is a promising approach for carbon waste revalorization [25,26] and sustainable chemistry [27]. After pyrolysis, elemental carbon can be easily retrieved to form a solid black powder [28]. The challenging point is the development of a platform for converting regenerated carbon from waste into industrially valuable products with the possibility of reaching an overall carbon-neutral cycle [29]. Calcium carbide can be considered an opportunity to return carbon back and close the carbon cycle.

Hydrolysis of calcium carbide results in gaseous acetylene, which can be easily integrated into industrial processes, providing a wide range of valuable chemicals [30,31,32,33,34]. In addition to the well-established large-scale manufacturing of acetylene, carbide is also used directly, skipping gaseous acetylene, in many fundamental processes [35,36,37,38,39,40,41,42,43,44,45,46,47,48,49,50,51]. Currently, calcium carbide is a valuable product of the chemical industry and is produced from natural limestone and fossil carbon [52,53,54,55,56,57,58]. The initial reagents in the manufacturing of carbide remained the same until, in 2010, Zhang and co-authors presented a powerful approach based on biochars [59], which were obtained by pyrolysis of biomass and successfully used instead of mined carbon. 

Considering carbon recycling opportunities, municipal solid waste (MSW) is one of the rich carbon sources of organically bound carbon produced each year in large amounts [60,61,62]. However, MSW recycling is accompanied by many challenges due to the layering and often heterogeneity of this type of waste [18]. Synthesis of calcium carbide from returned carbon from waste is a conceptually new approach developed here.

In this work, we developed a new approach using the product of pyrolysis as a starting source in the synthesis of calcium carbide instead of mined carbon. Pyrolyzed MSW, industrial waste and biomass sources and other carbonaceous materials (CMs) were used in carbide synthesis. As a co-reagent, calcium carbonate and calcium metal were used. The synthesis occurred inside a thermogravimetric apparatus in a special crucible. The yield of carbide was calculated according to the TG curves. Then, the same CMs were heated in a quartz tube with Ca metal to obtain carbide on a gram scale. According to the studied processes, waste carbon was used to produce CaC_2_ and acetylene and successfully employed for the synthesis of vinyl ethers—valuable monomers. To the best of our knowledge, for the first time, we discuss such a sustainable calcium carbide cycle with high carbon efficiency.

## 2. Results and Discussion

### 2.1. Concept of a Sustainable Ca Cycle Using Carbon-to-Acetylene Transformation

Converting carbon to acetylene is a useful process since a large variety of chemicals can be further obtained from acetylene using existing technologies. Calcium carbide is a powerful option for acetylene storage and on-demand generation upon a simple reaction with water:

CaC_2_ + 2H_2_O = Ca(OH)_2_ + C_2_H_2_

**Figure 2:**
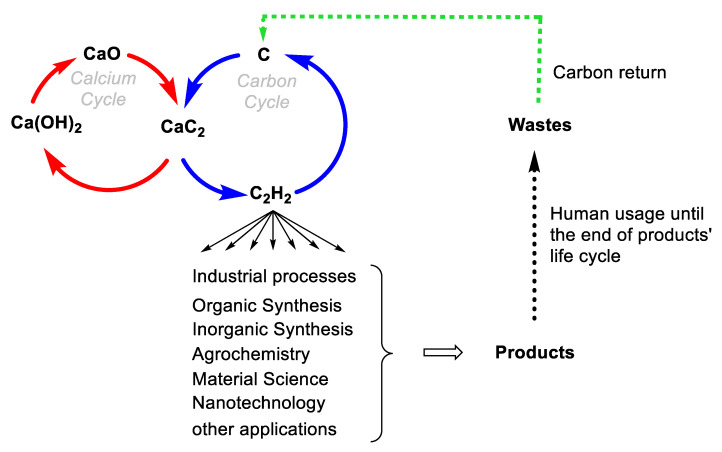
Closing loops for sustainable carbon recirculation.

Importantly, calcium hydroxide can be reused to produce calcium carbide again. In principle, a closed loop on calcium can be maintained (Figure 2). Pyrolysis of wastes to obtain carbon and synthesis of calcium carbide constitute another loop for the overall carbon to acetylene conversion (Figure 2, blue circle). By combining these two cycles, a certain amount of carbon can be kept in use without the need for fossil carbon mining and avoiding the negative scenario of increased carbon dioxide emissions. As a general idea, the possibility of a sustainable cycle can be considered using the combination of two closed loops (Figure 2).

### 2.2. Calcium Carbide Synthesis: Electric Arc at 2100 °C vs. Oven at 1500 °C

Industrial carbide manufacturing technology is based on electrodes that supply power to the furnace. In the mixture, heat transfer is significantly hindered [57,63,64], and considering the high enthalpy of carbide formation according to the equation, the process is carried out at high temperatures up to 2100–2200 °C to melt supplied portions of reagents immediately and reduce the reaction time. The decomposition of carbide at high temperatures is compensated by the fast rate of smelting and sufficient heating of outlying zones of the reaction mixture [65]. In total, the yield can reach 85% as a result of the optimized process.

When the reaction occurs inside a TG apparatus with facilitated heat transfer, milligrams of previously calcinated and fractionated reagents are usually used. This technique is a convenient approach for a comparative study of various carbon sources. However, the reaction itself should also be studied in larger quantities under regular conditions.

Here, we tested the reaction both in a TG spectrometer and in a regular quartz tube. The former process occurred in a ZrO_2_ crucible covered with a platinum cap. The latter reaction was carried out on a gram scale inside a vacuum oven at 1100 °C.

### 2.3. Carbon Content

Carbon content is an essential indicator of the quality of the initial carbonaceous raw materials. Obviously, the yield of calcium carbide depends on the available elemental carbon amount in the raw material. Note that carbon can be present in a sample both as elemental carbon and as a part of a compound. If a sample already contains elemental carbon or a compound that decomposes to form elemental carbon, the carbon will react with CaO. Otherwise, if carbon is bound in organic molecules, it may be unavailable for the synthesis of calcium carbide or the reaction efficiency would be much lower.

**Figure 3:**
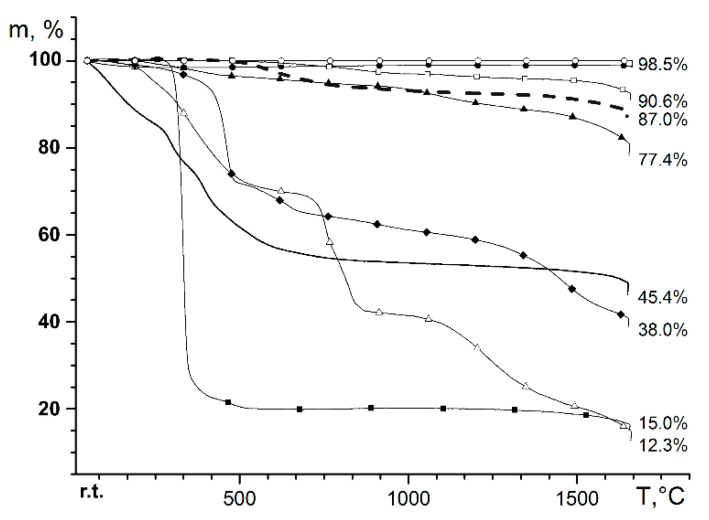
Weight loss in the selected samples according to TGA (m, %) and applied temperature program; notations of carbon materials: nanoglobular carbon and nanotubes, 98.5%; coke, 90.6%; CE2, 87%; carbon fiber, 77.4%; CE1, 45.4%; MSW, 38%; microcrystalline cellulose, 15%; humins, 12.3%.

Therefore, before the synthesis of carbide, the available carbon was determined by heating each sample from 0 °C up to 1680 °C in a ZrO_2_ crucible inside a TG apparatus under a He-atmosphere to detect weight loss. The upper-temperature limit was chosen to be related to the temperature of carbide synthesis. We also continued heating to check for further changes and, finally, stopped the curves at 1680 °C since no changes were detected. The carbon content was analyzed for the standard source—industrial coke; commercially available highly pure carbonaceous materials, such as carbon nanotubes (NT), nanoglobular carbon (NG), and carbon fiber (CF); residues after the pyrolysis of cation exchangers CE1 and CE2; MSW; humins (synthesized according to the literature procedure) [66] and microcrystalline cellulose (MCC) (Figure 3). NG and NT consisted almost entirely of carbon because their weight remained unchanged when heating. Mined coke usually used for the synthesis of carbide lost approximately 10% of its weight, presumably as a result of the decomposition of residual amounts of organic compounds. One of the selected standards, carbon fiber, showed a low carbon content (77.4%), which is most likely due to the incomplete decomposition of residual organic polymers during the previous treatment. To our surprise, one of the pyrolysis products of the cation exchange resin (CE2) lost only 13% in weight, approaching coke at this value. For another cation exchanger (CE1), the carbon content was lower (45.4%). A similar value was obtained for MSW (38%), which makes both materials promising for the synthesis of carbides. The lowest values were detected for humins (12.3%) and cellulose (15%). Both compounds are rich in carbon but the carbon is found in volatile organic or polymeric forms, resulting in low residual mass.

Thus, commercially available NG, NT, and coke were the best initial carbon sources in carbide synthesis. The high carbon content in MSW and CE2 is also promising, and all the sources were tested in the synthesis of calcium carbide inside a TG crucible. Humins were not chosen as a carbon source in carbide synthesis in TGA due to having the lowest carbon content (12.3%).

Then, the samples were burned under an oxygen atmosphere to determine the inorganic residue (see Supplementary Materials). The content of available elemental carbon was corrected and calculated as the difference between the residue after heating under an inert atmosphere and the residue under an oxygen atmosphere. All the experiments and calculations of loadings during the synthesis of calcium carbide were carried out related to the corrected residual amount.

### 2.4. Synthesis of Calcium Carbide in a TG Crucible

**Figure 4:**
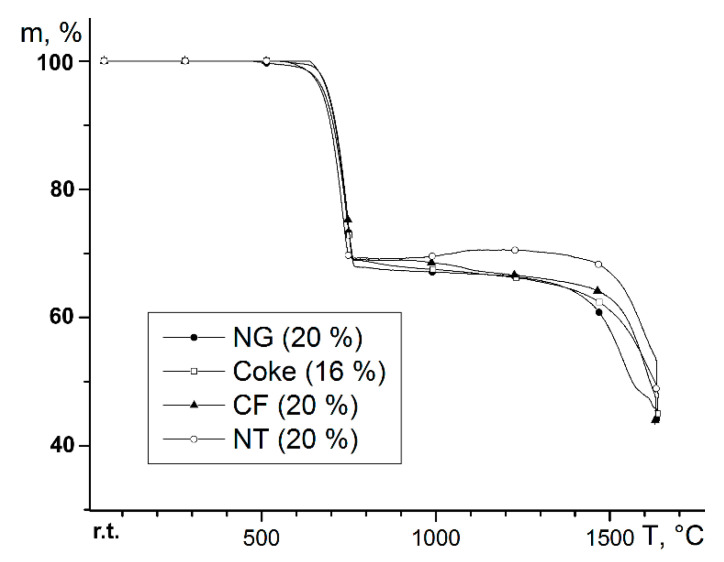
Weight loss in calcium carbide synthesis from commercial carbon sources: nanoglobules, coke, carbon fiber, and nanotubes according to TGA (m, %).

Graphite and calcium carbonate were chosen as standard reagents for the synthesis of calcium carbide. First, we varied the conditions of the synthesis (see Supplementary Materials). Then, under optimized conditions, different carbonaceous materials were tested in carbide synthesis. Calcium carbonate is completely decomposed upon reaching 1000 °C to carbon dioxide and calcium oxide, which further react with elemental carbon. Detailed optimization was previously carried out (molar ratios, reaction time, temperature) [59], and after checking the optimal conditions using calcium carbonate (see Supplementary Materials), all carbonaceous materials were tested in calcium carbide synthesis (Figure 4).

The reaction profiles were similar in each case. The first zone of weight loss was observed at 600–800 °C and corresponded to calcium carbonate decomposition with CO_2_ evolution (33% weight loss). Calcium carbonate decomposed completely in all cases in the same way (Equation (1)); some variations in weight loss were possible due to additional pyrolysis processes of carbonaceous materials (Figure 3). The second reaction corresponded to calcium carbide formation with CO evolution (Equation (2)) at 1500–1680 °C (NT, NG and CF—20%; and coke—16% weight loss). The reaction time depended on the temperature and heating rate and the process was terminated after completing the weight loss.

(1) $${\mathrm{CaCO}}_{3}\;\overset{600-800\;{}^{\circ}C}{\to }\;\mathrm{CaO}+{\mathrm{CO}}_{2}(\Delta m({\mathrm{CO}}_{2}))$$

(2) $$\mathrm{CaO}+3C\;\overset{1500-1680\;{}^{\circ}C}{\to }\;{\mathrm{CaC}}_{2}+{\mathrm{CO}}_{\;}(\Delta m(\mathrm{CO}))$$

**Figure 5:**
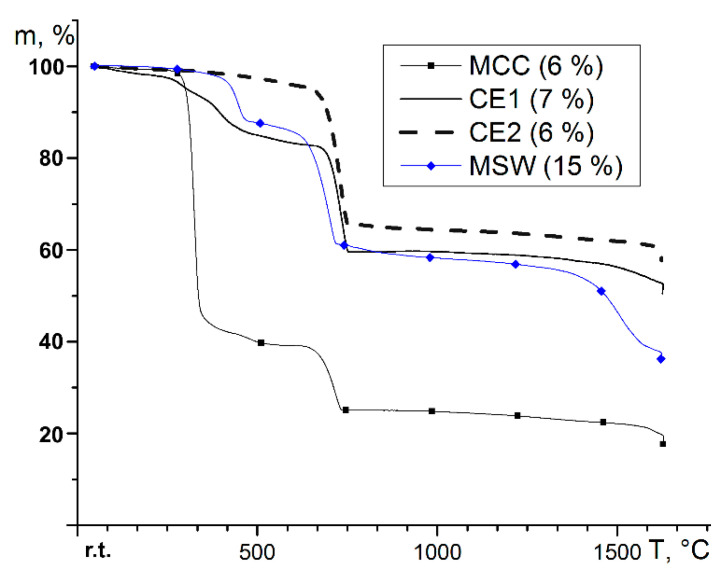
Weight loss in calcium carbide synthesis from waste materials: microcrystalline cellulose, cation exchangers, and municipal solid wastes.

Next, we tested carbonaceous materials as a carbon source in the reaction with calcium carbonate (Figure 5). Of course, the weight loss was lower in the CO zone for MCC and cation exchangers; however, the carbon content was much lower in those materials, therefore increasing the loading would lead to better results. Moreover, the weight loss in the case of MSW was similar to that of commercial materials (15%), therefore this result was very promising.

To calculate the yield of calcium carbide, the mass of CO released should be calculated since the formation of calcium carbide accompanied the release of only one volatile component (carbon oxide). Thus, the mass of CO released is an appropriate value related to the carbide amount (2). Furthermore, weight loss in the CO zone and carbide yield will be calculated. The total weight loss Δm(total) is a complicated value consisting of weight loss due to pyrolysis (Δm(pyr)), CO_2_ weight loss (Δm(CO_2_, total)) due to decomposition of calcium carbonate, and CO weight loss due to carbide formation (Δm(CO, total)) (3).

Δm(total) = Δm(pyr) + Δm(CO_2_, total) + Δm(CO, total) (3)

CO_2_ release starts and finishes at lower temperatures than carbide formation; thus, weight loss in the CO_2_ zone should be neglected.

At the same time, a part of a loaded CM was not pyrolyzed, and the inactive residue was determined after burning a sample of a CM under an oxygen atmosphere (designated inorganic residue); therefore, CM consists of a burned part and an inorganic residue (4).

m(CM, loaded) = m(burned residue) + m(inorg. residue)(4)

The pyrolyzed part can be calculated from the pyrolysis (m(CM, pyrolyzed)) and the part that reacted with CaO (C to CaC_2_) should be calculated according to (5) and (6).

m(C to CaC_2_) = m(C, loaded) − m(C, pyr) − m(inorg. residue) (5)

m(C, pyr) = m(CM, loaded) × Δm(pyr)(6)

Next, m(CO from CaC_2_) can be calculated according to Figure 4 and Figure 5.

m(CO from CaC_2_) = m(loaded) × Δm(CO, total) − m(CM, loaded) × Δm(pyr, CO zone) (7)

Then, comparing the CO mass from the reaction with the theoretical value and considering the purity (carbon amount) of the carbonaceous materials, the yield of calcium carbide can be calculated (Table 1; detailed Table S1 is in Supplementary Materials).

As expected, the yields of calcium carbide from commercial sources were very high (97–99%). Cation exchangers show moderate results (27 and 24%), considering the high carbon content. However, the exchangers were presented as small solid balls and were hard to grind. Indeed, grinding is fundamentally important for the reaction, therefore we assume that with dedicated industrial grinding, the carbide yield can be significantly improved. MSW was used as a fine powder and showed excellent results (99%). The yield of carbide derived from MCC was moderate (54%); however, the reaction is of academic interest only due to the very low carbon content available for the reaction in the initial carbonaceous material.

The purity of the carbide was confirmed by means of gas chromatography using a column based on molecular sieves for acetylene detection. The synthesized powders were mixed with water in a vial and an aliquot of the gas was injected into a chromatograph (no other gases were detected during the analysis).

### 2.5. Gram-Scale Synthesis of Calcium Carbide

The synthesis of carbides in a TG apparatus is a convenient technique for a mechanistic study. Uniform rapid heating of a reaction mixture ensures the high performance of the reaction. However, these conditions are difficult to achieve in real synthesis on a larger scale. In addition, the mass of the resulting carbide in a TG analyzer was very small (several milligrams): this amount is not enough for a complete investigation and even isolated yield determination, and during the transfer of such amounts, the synthesized carbide is rapidly hydrolyzed.

Therefore, the reaction with the tested carbonaceous materials (Table S2) in gram quantities was also evaluated. The reaction involving calcium metal with carbonaceous materials was selected since no CO gas evolution takes place and no carbon is lost during the reaction. In this work, there is no discussion or call for replacing the standard method of calcium carbide manufacturing from lime and coke with a method from calcium metal. Of course, calcium metal is more expensive than lime and even the desired carbide. This method of obtaining carbide (from calcium and carbon) was used in this work only as a test and verification of the presence (and content) of carbon in carbonaceous materials since it is not possible to implement gram synthesis according to the classical scheme at 2200 °C in the laboratory.

**Scheme 1:**
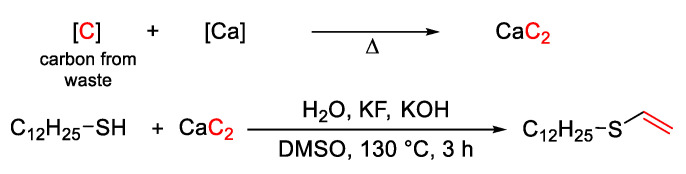
Synthesis of thiovinyl ether from calcium carbide.

Pieces of calcium metal 2–4 mm in size were mixed with the carbonaceous materials in a 1.1:2.0 ratio. The reaction of CaC_2_ preparation occurred in a vacuum oven in quartz glass at 1100 °C for an hour (Scheme 1). The oven was cooled after the process under an argon atmosphere. No optimization was performed since this reaction is fundamentally different and this work is a separate investigation (in progress in our lab).

Determination of the content of calcium carbide in the final product (the yield) is a challenging task due to the volatile nature of acetylene. In the case of a standard volumetric approach, the accuracy of measurements may be very low. Transfer of carbide from a reaction vessel is accompanied by hydrolysis. Therefore, the yields were determined according to the nucleophilic addition to acetylene (i.e., thiovinylation). Thiovinylation, as a special case of vinylation, provides quantitate yields in the case of some thiols and completely converts gaseous acetylene into the corresponding thiovinyl ethers (Scheme 1, see also Supplementary Materials and reference [35] for more information), which can be easily detected by NMR. Dodecanethiol was selected as a model thiol for this reaction.

The selected commercial carbide sample showed 75% calcium carbide purity in the chemical. After that, the reaction with the same thiol was carried out by varying the thiol/carbide ratio, and the final thiol/vinyl ether ratio was determined according to the ^1^H NMR spectrum. As a result, a calibration curve was created (see Supplementary Materials). The tested CM-derived carbides were studied under the same conditions, so the determination of the purities was precise enough due to the same conditions. The best result was obtained with pure commercial graphite (89%) since the content and purity of available carbon were the highest. Very promising results were obtained with CE2 (54%); moreover, using MSW as a carbon source in the synthesis of carbide is also possible (37% yield). The other CE1 showed a poor purity of 23%.

Comparing the yields of carbides obtained by different methods, it can be concluded that the yield of carbide depends on the amount of available carbon in a CM. Since the reactions for carbide synthesis are completely different (the first originates from a CM and calcium carbonate, and the second one is from a CM and calcium metal), different CMs provided various yields. When the reaction mixture is heated, residual organic impurities decompose, and the carbide yield depends on the carbon content in the mixture at a current temperature. In the case of MCC, the temperature of 1100 °C is insufficient to decompose the carbon-containing compounds, and the existing carbon-containing compounds decompose at a higher temperature, which gives a yield upon reaction with calcium oxide. At the same time, the decomposition of calcium carbonate produces carbon dioxide, which can react with carbon-containing compounds in the mixture, thereby reducing the yield. Either the formed calcium oxide can block some compounds due to an alkaline nature or by reacting and converting the required carbon into refractory components.

## 3. Materials and Methods

Materials. Calcium carbonate, calcium carbide (granulated, technical), potassium hydroxide, calcium metal, dodecanethiol, DMSO were supplied by Sigma-Aldrich and used as received without any further purification. Potassium fluoride was received from a local company Vecton and was ground and dried at 170 °C for 24 h. 

Synthesis of Calcium Carbide in the TG Apparatus (Netzsch). A mixture of a carbonaceous material and calcium carbonate was placed in a crucible and placed in the apparatus. After heating, the mixture was kept under dry atmosphere and used in further experiments. 

Synthesis of Calcium Carbide in an Oven. Each mixture of calcium metal and carbonaceous material in appropriate ratio was loaded into a quartz tube and placed into a vacuum oven. The oven was heated to 1100 °C for an hour. After the reaction, the oven was cooled to room temperature and the residue was placed in a sealed vial and then used.

Volumetric Investigations. A round-bottom flask equipped with a pressure-compensated dropping funnel and an outlet was charged with 25 mg of commercial CaC_2_. A dropping funnel was filled with 25 mL of water and sealed with a stopper. A silicone hose was connected to the outlet of the flask, the end of which was placed underwater in an inverted 10 mL measuring cylinder completely filled with water. Then, while stirring, an excess of water (10 mL) was added dropwise to the flask, while acetylene was quickly released, the excess of which went through a hose into a graduated cylinder, which was used to determine the volume of evolved gas.

Characterization Methods. NMR spectra were recorded using Bruker 400 MHz NMR spectrometer WB Avance III (Germany). Deuterated solvents (chloroform-*d* and DMSO-*d_6_*) were purchased from Cambridge Isotope Laboratories, Inc., UK. Chloroform-*d* was previously passed through a column with Al_2_O_3_ to remove the traces of the acid and stored under molecular sieves. 

Chromatographic Identifications. A sample of commercially available calcium carbide (1 mg or less) was placed into a vial (1.8 mL). Using a syringe, a drop of water was injected into a vial. After the reaction, a sample of gas in vial was taken in a gas syringe and injected into a “Kristall” gas-chromatograph. A peak of acetylene was identified. The same procedure was carried out with synthesized calcium carbide samples using new gas syringe each time. The retention time of acetylene peak was the same as in the case of using commercially available calcium carbide. Note, gas syringe after dosing should be kept open during night. Every determination must be started with a blank sample of air to check the residual amounts of acetylene gas in a syringe. 

## 4. Conclusions

A series of carbonaceous materials were tested as a carbon source in the synthesis of calcium carbide according to a common procedure. The sustainable potential for carbon reuse was studied for MSW and cation exchangers. Excellent yields were obtained in the reaction of MSW with calcium carbonate towards the formation of calcium carbide (99%).

Practical applications should be considered taking into account that pyrolysis is applicable to revalorize various types of waste. However, black carbon-containing powder obtained after pyrolysis is currently often disposed of. We found that this residue can be successfully utilized as a carbon source in the synthesis of calcium carbide. Turning carbon wastes into acetylene is a promising general opportunity to achieve a sustainable carbon cycle (Figure 2).

Overall, we believe that the sustainable potential of calcium carbide should be rethought and that the conversion of carbon-containing wastes to acetylene may be a useful route.

## Figures and Tables

**Table 1:** Calcium carbide yield data in TGA.

CM	C, % ^a^	Δm_(CO,total)_, % (mg) ^b^	Δm_(pyr,CO zone)_, % (mg) ^c^	m_(CO)_, mg ^d^	Yield, % ^e^
CF	67.9	19.6 (1.80)	9.8 (0.28)	1.51	99
NT	98.0	20.2 (1.50)	2.0 (0.04)	1.46	97
Coke	66.4	15.8 (1.69)	4.8 (0.15)	1.54	99
NG	98.0	20.0 (1.71)	0.5 (0.01)	1.70	98
CE1	40.4	6.7 (0.64)	6.3 (0.27)	0.37	27
CE2	86.2	6.0 (0.53)	4.5 (0.12)	0.41	24
MSW	28.5	15.0 (1.62)	9.0 (0.47)	1.14	99
MCC	12.7	6.1 (0.74)	3.7 (0.31)	0.44	54

^a^ Reactive carbon content, determined according to pyrolysis, burning in an oxygen atmosphere, and inorganic residue determination. ^b^ Total weight loss as a result of CO emission. The first values are from Figure 4 and Figure 5 and the second in brackets are calculated from the loadings (see Supplementary Materials). ^c^ Weight loss in the CO zone as a result of pyrolysis. The first values are from Figure 3 and the second values in brackets are calculated from the loadings (see Supplementary Materials). ^d^ The real masses of CO released, calculated according to (7). The theoretical values are listed in Supplementary Materials. ^e^ The yields of calcium carbide considering the carbon content in carbonaceous materials according to burning and pyrolysis data.

## Supplementary Materials

The following supporting information can be downloaded at: https://www.mdpi.com/article/10.3390/ijms231911828/s1.

## References

[1] Smieja J.M., Babcock K.E. (2017). The intersection of green chemistry and Steelcase’s path to circular economy. Green Chem. Lett. Rev..

[2] Linder M. (2017). Ripe for disruption: Reimagining the role of green chemistry in a circular economy. Green Chem. Lett. Rev..

[3] Lakshmidevi J., Ramesh Naidu B., Avula S.K., Majhi A., Chia P.W., Al-Harrasi A., Venkateswarlu K. (2022). A waste valorization strategy for the synthesis of phenols from (hetero)arylboronic acids using pomegranate peel ash extract. Green Chem. Lett. Rev..

[4] Magagula L.P., Masemola C.M., Ballim M.A., Tetana Z.N., Moloto N., Linganiso E.C. (2022). Lignocellulosic Biomass Waste-Derived Cellulose Nanocrystals and Carbon Nanomaterials: A Review. Int. J. Mol. Sci..

[5] Nomanbhay S., Hussein R., Ong M.Y. (2018). Sustainability of biodiesel production in Malaysia by production of bio-oil from crude glycerol using microwave pyrolysis: A review. Green Chem. Lett. Rev..

[6] Alvarez-Gallego C.J., Fdez-Güelfo L.A., Romero Aguilar M.d.l.A., Romero García L.I. (2015). Thermochemical Pretreatments of Organic Fraction of Municipal Solid Waste from a Mechanical-Biological Treatment Plant. Int. J. Mol. Sci..

[7] Elkhalifa S., Mariyam S., Mackey H.R., Al-Ansari T., McKay G., Parthasarathy P. (2022). Pyrolysis Valorization of Vegetable Wastes: Thermal, Kinetic, Thermodynamics, and Pyrogas Analyses. Energies.

[8] Jha S., Nanda S., Acharya B., Dalai A.K. (2022). A Review of Thermochemical Conversion of Waste Biomass to Biofuels. Energies.

[9] Sato K., Yamamoto A., Dyballa M., Hunger M. (2022). Molecular adsorption by biochar produced by eco-friendly low-temperature carbonization investigated using graphene structural reconfigurations. Green Chem. Lett. Rev..

[10] John K.I., Omorogie M.O. (2022). Biomass-based hydrothermal carbons for catalysis and environmental cleanup: A review. Green Chem. Lett. Rev..

[11] Stahel W.R. (2016). The circular economy. Nature.

[12] Korhonen J., Honkasalo A., Seppälä J. (2018). Circular Economy: The Concept and its Limitations. Ecol. Econ..

[13] Geissdoerfer M., Savaget P., Bocken N.M.P., Hultink E.J. (2017). The Circular Economy—A new sustainability paradigm?. J. Clean. Prod..

[14] Ghisellini P., Cialani C., Ulgiati S. (2016). A review on circular economy: The expected transition to a balanced interplay of environmental and economic systems. J. Clean. Prod..

[15] Kirchherr J., Reike D., Hekkert M. (2017). Conceptualizing the circular economy: An analysis of 114 definitions. Resour. Conserv. Recycl..

[16] Barra A., Nunes C., Ruiz-Hitzky E., Ferreira P. (2022). Green Carbon Nanostructures for Functional Composite Materials. Int. J. Mol. Sci..

[17] Abis M., Bruno M., Kuchta K., Simon F.-G., Grönholm R., Hoppe M., Fiore S. (2020). Assessment of the Synergy between Recycling and Thermal Treatments in Municipal Solid Waste Management in Europe. Energies.

[18] Zhao X., Korey M., Li K., Copenhaver K., Tekinalp H., Celik S., Kalaitzidou K., Ruan R., Ragauskas A.J., Ozcan S. (2022). Plastic waste upcycling toward a circular economy. Chem. Eng. J..

[19] Zhang X., Liu Y. (2022). Circular economy is game-changing municipal wastewater treatment technology towards energy and carbon neutrality. Chem. Eng. J..

[20] Andersen M.S. (2007). An introductory note on the environmental economics of the circular economy. Sustainability Sci..

[21] Peters G.P., Weber C.L., Guan D., Hubacek K. (2007). China’s Growing CO2 EmissionsA Race between Increasing Consumption and Efficiency Gains. Environ. Sci. Technol..

[22] Lieder M., Rashid A. (2016). Towards circular economy implementation: A comprehensive review in context of manufacturing industry. J. Clean. Prod..

[23] Blomsma F., Brennan G. (2017). The Emergence of Circular Economy: A New Framing Around Prolonging Resource Productivity. J. Ind. Ecol..

[24] Kalmykova Y., Sadagopan M., Rosado L. (2018). Circular economy—From review of theories and practices to development of implementation tools. Resour. Conserv. Recycl..

[25] Pichon A. (2012). A promising pyrolysis. Nat. Chem..

[26] Qureshi M.S., Oasmaa A., Pihkola H., Deviatkin I., Tenhunen A., Mannila J., Minkkinen H., Pohjakallio M., Laine-Ylijoki J. (2020). Pyrolysis of plastic waste: Opportunities and challenges. J. Anal. Appl. Pyrolysis.

[27] Metlyaeva S.A., Rodygin K.S., Lotsman K.A., Samoylenko D.E., Ananikov V.P. (2021). Biomass- and calcium carbide-based recyclable polymers. Green Chem..

[28] Zhang S., Jiang S.-F., Huang B.-C., Shen X.-C., Chen W.-J., Zhou T.-P., Cheng H.-Y., Cheng B.-H., Wu C.-Z., Li W.-W. (2020). Sustainable production of value-added carbon nanomaterials from biomass pyrolysis. Nature Sust..

[29] Martínez J.D., Cardona-Uribe N., Murillo R., García T., López J.M. (2019). Carbon black recovery from waste tire pyrolysis by demineralization: Production and application in rubber compounding. Waste Manag..

[30] Schobert H. (2014). Production of Acetylene and Acetylene-based Chemicals from Coal. Chem. Rev..

[31] Trotuş I.-T., Zimmermann T., Schüth F. (2014). Catalytic Reactions of Acetylene: A Feedstock for the Chemical Industry Revisited. Chem. Rev..

[32] Brand J.P., Waser J. (2012). Electrophilic alkynylation: The dark side of acetylene chemistry. Chem. Soc. Rev..

[33] Trofimov B.A., Schmidt E.Y. (2018). Acetylenes in the Superbase-Promoted Assembly of Carbocycles and Heterocycles. Acc. Chem. Res..

[34] Trofimov B.A., Gusarova N.K. (2007). Acetylene: New prospects of classical reactions. Russ. Chem. Rev..

[35] Rodygin K.S., Ananikov V.P. (2016). An efficient metal-free pathway to vinyl thioesters with calcium carbide as the acetylene source. Green Chem..

[36] Gao L., Li Z. (2020). Synthesis of Aromatic Terminal Allenes and Aliphatic Terminal Alkynes from Hydrazones Using Calcium Carbide as an Acetylene Source. Org. Chem. Front..

[37] Lu H., Li Z. (2019). Palladium-Catalyzed One-Pot Four-Component Synthesis of β-Cyano-α,β-unsaturated Ketones Using Calcium Carbide as an Acetylene Source and Potassium Hexacyanoferrate(II) as an Eco-Friendly Cyanide Source. Adv. Synth. Catal..

[38] Hosseini A., Schreiner P.R. (2019). Synthesis of Exclusively 4-Substituted β-Lactams through the Kinugasa Reaction Utilizing Calcium Carbide. Org. Lett..

[39] Turberg M., Ardila-Fierro K.J., Bolm C., Hernández J.G. (2018). Altering Copper-Catalyzed A3 Couplings by Mechanochemistry: One-Pot Synthesis of 1,4-Diamino-2-butynes from Aldehydes, Amines, and Calcium Carbide. Angew. Chem. Int. Ed..

[40] Lu H., Li Z. (2020). Synthesis of 1,2,3-Triazolyl-Based Ketoximes Using Calcium Carbide as an Acetylene Source. Eur. J. Org. Chem..

[41] Yang P.-W., Liu X.-X., Li X.-Q., Wei M.-X. (2021). Transition metal-free and solvent-free calcium carbide promotes the formation of β-keto sulfoxide from acyl chloride and DMSO. Org. Chem. Front..

[42] Liu Z., Li Z. (2021). Synthesis of 1,3-Diynes Using Calcium Carbide as an Alkyne Source. Eur. J. Org. Chem..

[43] Ma X., Li Z. (2021). Synthesis of Diarylethynes from Aryldiazonium Salts by Using Calcium Carbide as an Alkyne Source in a Deep Eutectic Solvent. Synlett.

[44] Fu R., Lu Y., Yue G., Wu D., Xu L., Song H., Cao C., Yu X., Zong Y. (2021). Direct Synthesis of 3-Coumaranones with Calcium Carbide as an Acetylene Source. Org. Lett..

[45] Rodygin K.S., Samoylenko D.E., Seitkalieva M.M., Lotsman K.A., Metlyaeva S.A., Ananikov V.P. (2022). Generation, regeneration, and recovery of Cu catalytic system by changing the polarity of electrodes. Green Chem..

[46] Lebedev A.N., Rodygin K.S., Mironenko R.M., Saybulina E.R., Ananikov V.P. (2022). Metal-Catalyzed Chemical Activation of Calcium Carbide: New Way to Hierarchical Metal/Alloy-on-Carbon Catalysts. J. Catal..

[47] Rodygin K.S., Ledovskaya M.S., Voronin V.V., Lotsman K.A., Ananikov V.P. (2021). Calcium Carbide: Versatile Synthetic Applications, Green Methodology and Sustainability. Eur. J. Org. Chem..

[48] Voronin V.V., Ledovskaya M.S., Rodygin K.S., Ananikov V.P. (2020). Examining the vinyl moiety as a protecting group for hydroxyl (-OH) functionality under basic conditions. Org. Chem. Front..

[49] Rodygin K.S., Lotsman K.A., Ananikov V.P. (2020). Calcium Carbide Looping System for Acetaldehyde Manufacturing from Virtually any Carbon Source. ChemSusChem.

[50] Rodygin K.S., Bogachenkov A.S., Ananikov V.P. (2018). Vinylation of a Secondary Amine Core with Calcium Carbide for Efficient Post-Modification and Access to Polymeric Materials. Molecules.

[51] Hosseini A., Schreiner P.R. (2020). Direct Exploitation of the Ethynyl Moiety in Calcium Carbide Through Sealed Ball Milling. Eur. J. Org. Chem..

[52] Morehead J.T., de Chalmot G. (1896). The Manufacture of Calcium Carbide. J. Am. Chem. Soc..

[53] Li M., Chen S., Dai H., Zhao H., Jiang B. (2021). Experimental Investigation on the Mass Diffusion Behaviors of Calcium Oxide and Carbon in the Solid-State Synthesis of Calcium Carbide by Microwave Heating. Molecules.

[54] Wang R., Liu Z., Ji L., Guo X., Lin X., Wu J., Liu Q. (2016). Reaction kinetics of CaC_2_ formation from powder and compressed feeds. Front. Chem. Sci. Eng..

[55] Gong X.-z., Zhang J.-q., Wang Z., Wang D., Liu J.-h., Jing X.-d., Qian G.-y., Wang C. (2021). Development of calcium coke for CaC_2_ production using calcium carbide slag and coking coal. Int. J. Miner. Metall. Mater..

[56] Xiong W., Chen X., Wang Q., Gao M., Zheng D., Mai Y., Hu W. (2020). Heterogeneous Phases Reaction Equilibrium in an Oxy-Thermal Carbide Furnace. ChemEngineering.

[57] Xu Q., Li Y., Deng S., He Y.-L., Li L., Yu H. (2019). Modeling of Multiprocess Behavior for Feedstock-Mixed Porous Pellet: Heat and Mass Transfer, Chemical Reaction, and Phase Change. ACS Sust. Chem. Eng..

[58] Li Z., Liu Z., Wang R., Guo X., Liu Q. (2018). Conversion of bio-char to CaC_2_ at low temperatures-morphology and kinetics. Chem. Eng. Sci..

[59] Li G., Liu Q., Liu Z., Zhang Z.C., Li C., Wu W. (2010). Production of Calcium Carbide from Fine Biochars. Angew. Chem. Int. Ed..

[60] Venkata M.S., Nikhil G.N., Chiranjeevi P., Reddy N.C., Rohit M.V., Kumar A.N., Sarkar O. (2016). Waste biorefinery models towards sustainable circular bioeconomy: Critical review and future perspectives. Bioresour. Technol..

[61] Aghbashlo M., Tabatabaei M., Soltanian S., Ghanavati H., Dadak A. (2019). Comprehensive exergoeconomic analysis of a municipal solid waste digestion plant equipped with a biogas genset. Waste Manag..

[62] Miezah K., Obiri-Danso K., Kádár Z., Fei-Baffoe B., Mensah M.Y. (2015). Municipal solid waste characterization and quantification as a measure towards effective waste management in Ghana. Waste Manag..

[63] Li R., Ma S., Ma H., Liu S., Wang H. (2020). Numerical simulation of heat transfer and chemical reaction of CaO-C porous pellets in the reaction layer of calcium carbide furnace. Appl. Therm. Eng..

[64] Zhang X.-K., Tong Z.-X., He Y.-L., Hu X. (2021). Influence of feed architecture on heat and mass transfer in calcium carbide electric furnace. Int. J. Heat Mass Transf..

[65] Ji L., Liu Q., Liu Z. (2014). Thermodynamic Analysis of Calcium Carbide Production. Ind. Eng. Chem. Res..

[66] Chernysheva D.V., Chus Y.A., Klushin V.A., Lastovina T.A., Pudova L.S., Smirnova N.V., Kravchenko O.A., Chernyshev V.M., Ananikov V.P. (2018). Cover Feature: Sustainable Utilization of Biomass Refinery Wastes for Accessing Activated Carbons and Supercapacitor Electrode Materials. ChemSusChem.

