# Supplementary material for: Towards Sustainable Carbon Return from Waste to Industry via C2-Type Molecular Unit
Source: Int J Mol Sci. 2022 Oct 5;23(19):11828. doi: 10.3390/ijms231911828 (PMC9569557; doi:10.3390/ijms231911828)
Supplement: Supplementary file 1 [file ijms-23-11828-s001.zip › ijms-1930071-supplementary.pdf]

Supporting Information
For the article

Towards Sustainable Carbon Return from Waste to Industry via C_2-Type Molecular Unit

Konstantin S. Rodygin,[a] Kristina A. Lotsman,[a] Dmitriy E. Samoylenko,[a] Viktor M. Kuznetsov,[a] Valentine P. Ananikov,[a,b]*

[a] Konstantin Rodygin, Kristina Lotsman, Dmitriy Samoylenko, Viktor Kuznetsov
Saint Petersburg State University
Saint Petersburg, Universitetskiy pr. 26, 198504, Russia

[b] Valentine Ananikov
N. D. Zelinsky Institute of Organic Chemistry
Moscow, Leninsky pr. 47, 119991, Russia
E-mail: val@ioc.ac.ru; http://AnanikovLab.ru

Contents

1. The synthesis of calcium carbide from calcium carbonate 3

2. Detailed calculations of the calcium carbide yield in the TG apparatus. 4

3. Combustion data of the initial carbonaceous materials. 5

4. The synthesis of calcium carbide in an oven. 6

4.1. General procedure for the synthesis. 6

4.2. General procedure for volumetric investigations of commercial CaC_2 6

4.3. General procedure for the determination of calcium carbide yield by means of the thiovinylation reaction.. 6

4.4. The calibration data for the synthesized carbide in an oven. 6

4.5. Determination of carbide yield after the reaction in an oven. 7

5. NMR spectra. 9

1. The synthesis of calcium carbide from calcium carbonate

The procedure. Samples with different ratios of calcium carbonate and graphite were created and heated in ZrO_2 crucibles up to 1680 °C until full completion of the weight loss. The $CaCO_3$:graphite 1:3 ratio was the best (the maximum weight loss) and was further used in the synthesis of calcium carbide. The reaction consisted of three zones.

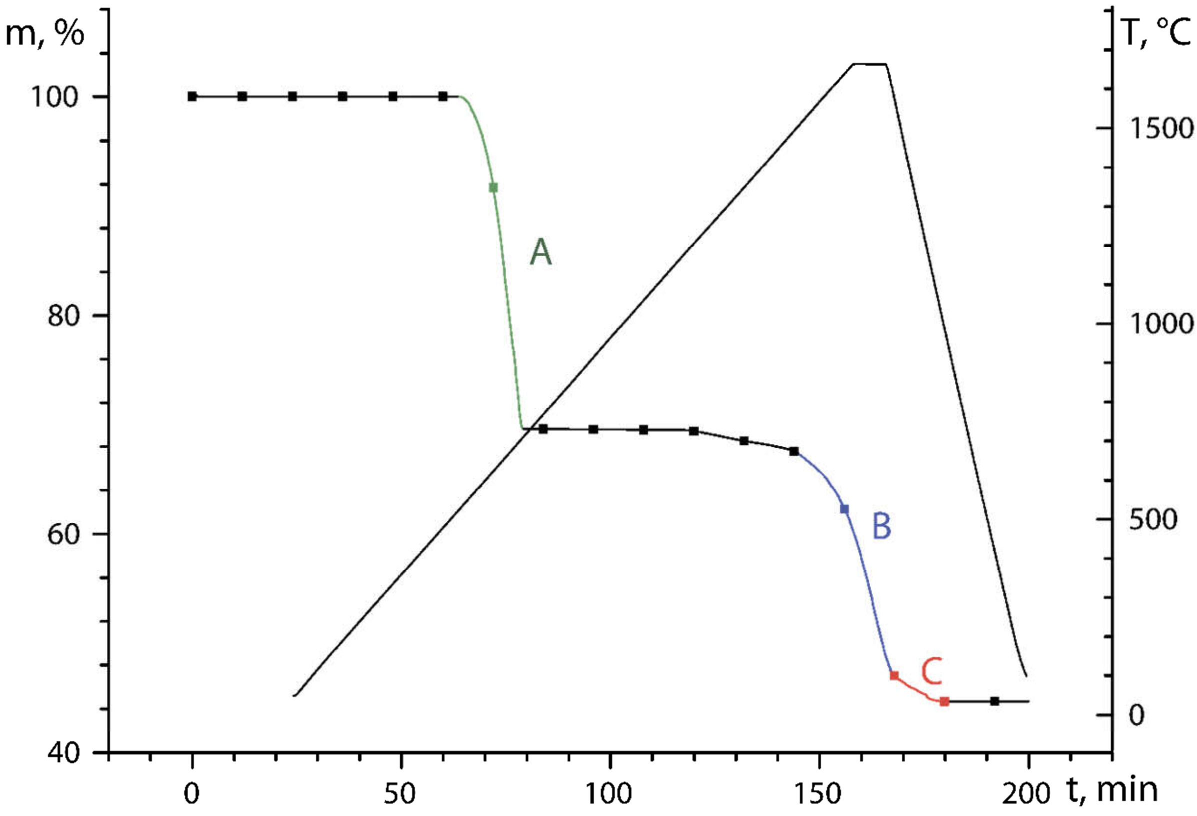

Figure S1. Optimization of the synthesis of calcium carbide from calcium carbonate and graphite.

The reaction zones are as follows:

A. Calcium carbonate decomposition to calcium oxide and carbon dioxide.

$CaCO_3 = CaO + CO_2$

B. Calcium carbide formation from calcium oxide and graphite.

$CaO + 3C = CaC_2 + CO$

C. Calcium carbide decomposition.

$CaC_2 + 2CaO = 3Ca + 2CO$

2. Detailed calculations of the calcium carbide yield in the TG apparatus.

Table S1.

CM	Loading, mg	Ratio ($CaCO_3$/CM[a])		m(CM), mg	m ($CaCO_3$), mg	Δm(CO, total),[b] %	Δm(CO, total), mg	Δm(pyr,CO zone),[c] %	Δm(pyr,CO zone), mg	Δm(CO), mg	C content, pyrolysis, %	m CO theory, mg	CaC_2 yield,[d] %	Inorg. residue,[e] %	C content, total,[f] %	m CO theory,[g] mg	CaC_2 yield, %
CF	9.17	100	46	2.89	6.28	19.6	1.80	9.8	0.28	1.51	77.4	1.76	86	9.5	67.9	1.53	99
NT	7.46	100	38.2	2.06	5.40	20.2	1.50	2.0	0.04	1.46	98.5	1.51	97	0.5	98.0	1.51	97
Coke	10.73	50	19.6	3.02	7.71	15.8	1.69	4.8	0.15	1.54	90.6	2.16	72	24.2	66.4	1.56	99
NG	8.56	50	19	2.36	6.20	20.0	1.71	0.5	0.01	1.70	98.5	1.74	98	0.5	98.0	1.74	98
CE1	9.60	100	81.5	4.31	5.29	6.7	0.64	6.3	0.27	0.37	45.4	1.48	25	5.0	40.4	1.36	27
CE2	8.90	100	44.5	2.74	6.16	6.0	0.53	4.5	0.12	0.41	87	1.72	24	0.8	86.2	1.72	24
MSW	10.80	100	93.2	5.21	5.59	15.0	1.62	9.0	0.47	1.14	38	1.56	73	9.5	28.5	1.16	99
MCC	12.22	50	104	8.25	3.97	6.1	0.74	3.7	0.31	0.44	15	1.11	39	2.3	12.7	0.82	54

a - including pyrolysis; b – according to Fig. 4 and Fig. 5; c – according to Fig. 3 at the same temperatures as Δm(CO, total); d – calculated by carbon including pyrolysis; e – according to Fig. S2; f – including pyrolysis and burning; g – calculated by deficiency (for NT, NG and CE2 for carbonate, for the rest of carbon).

3. Combustion data of the initial carbonaceous materials.

A sample of a material was placed into a crucible and heated at 1000 °C in an oxygen atmosphere inside the TG analyzer with continuous weight loss detection.

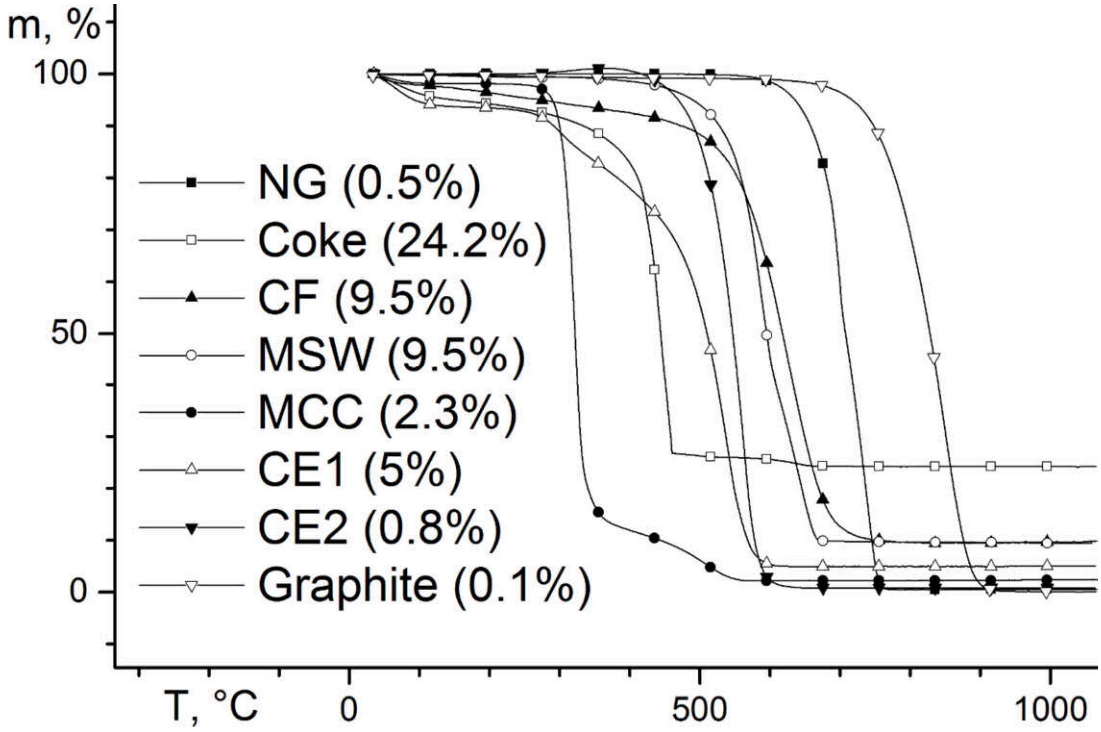

Figure S2. Combustion of the initial carbonaceous materials. The inorganic incombustible residue is in brackets.

4. The synthesis of calcium carbide in an oven.

4.1. General procedure for the synthesis.

Each mixture (Table S2) was loaded into a quartz glass and placed into a vacuum oven. The oven was heated to 1100 °C for an hour. After the reaction, the oven was cooled to rt, and the residue was placed in a sealed vial and then used.

Table S2. Preparation of mixtures for CaC_2 synthesis[a]

CM	Carbon content,[b] %	M(CM), g	M(Ca), g
Graphite	100	1.50	2.75
CEI	45.4	0.82	0.69
CEII	87	0.43	
MSW	38	0.99	
MCC	15	2.50	

a - the ratio of calcium to carbon is 1.1: 2 (mol); b – according to pyrolysis.

4.2. General procedure for volumetric investigations of commercial CaC_2

A round bottom flask equipped with a pressure-compensated dropping funnel and an outlet was charged with 25 mg of commercial CaC_2. A dropping funnel was filled with 25 ml of water and sealed with a stopper. A silicone hose was connected to the outlet of the flask, the end of which was placed under water in an inverted 10 ml measuring cylinder completely filled with water. Then, while stirring, an excess of water (10 ml) was added dropwise to the flask, while acetylene was quickly released, the excess of which went through a hose into a graduated cylinder, which was used to determine the volume of evolved gas.

According to theoretical calculations, 8.74 ml of acetylene should be released from 25 mg of calcium carbide. In the course of three runs, 6.6, 6.5, and 6.5 ml of the gas were captured, which corresponds to carbide contents of 75.5, 74.4 and 74.4%, respectively. Thus, averaging the obtained values, it was assumed that the content of pure CaC_2 in the commercial sample was 75%.

4.3. General procedure for the determination of calcium carbide yield by means of the thiovinylation reaction.

2 mL of DMSO, 187 µL (0.78 mmol) of diethylthiol, 52 mg (0.858 mmol) of KF, 50 mg (0.94 mmol) of KOH and an appropriate amount of calcium carbide were charged into a reaction tube. The tube was purged with argon, water (60 µL) was added, and the tube was sealed. The mixture was heated to 130 °C for 3 hours under stirring. Then, the mixture was cooled to rt, and an aliquot of 200 µL was diluted with 400 µL of DMSO-d6. The yield of VE was determined according to the intensity of the methyl group of thioVE and the initial thiol.

$$\text{Yield (thioVE)} = \frac{3}{I_{CH3}} \cdot 100\%.$$

4.4. The calibration data for the synthesized carbide in an oven.

The calibration data are listed in Table S3.

Table S3. The calibration data.

№	CaC_2, mg	Pure CaC_2, mg	Yield (thioVE), %
1	0	0	0
2	13.4	10	9.8
3	26.7	20	18.1
4	40.1	30	25.0
5	53.5	40	36.9

6	66.9	50	48.2
7	80.2	60	78.5

According to the obtained data, the calibration curve was created (Figure S3).

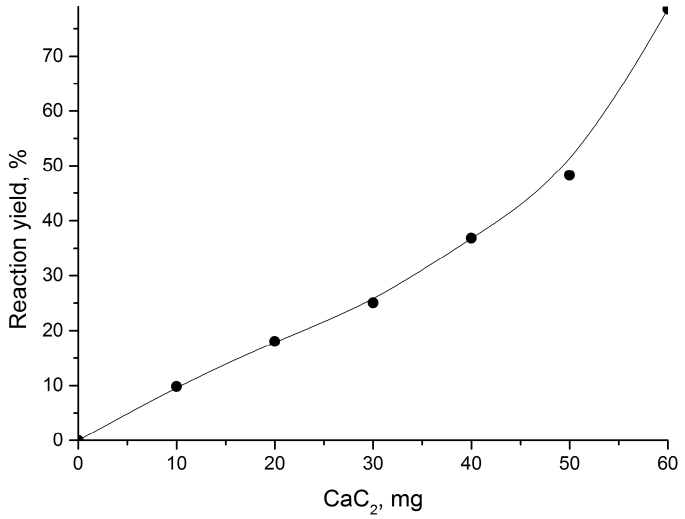

Figure S3. The calibration curve: yield of thioVE/loading of calcium carbide.

4.5. Determination of carbide yield after the reaction in an oven.

To determine the purity of the calcium carbide obtained, we used the calibration curve and the yields of the thioVE obtained under the same conditions with a load of 60 mg of the carbide. The experimental procedure is described elsewhere.[1] According to the yield of thioVE, we calculated the amount of carbide and calculated its content in the mixture (Figure S4).

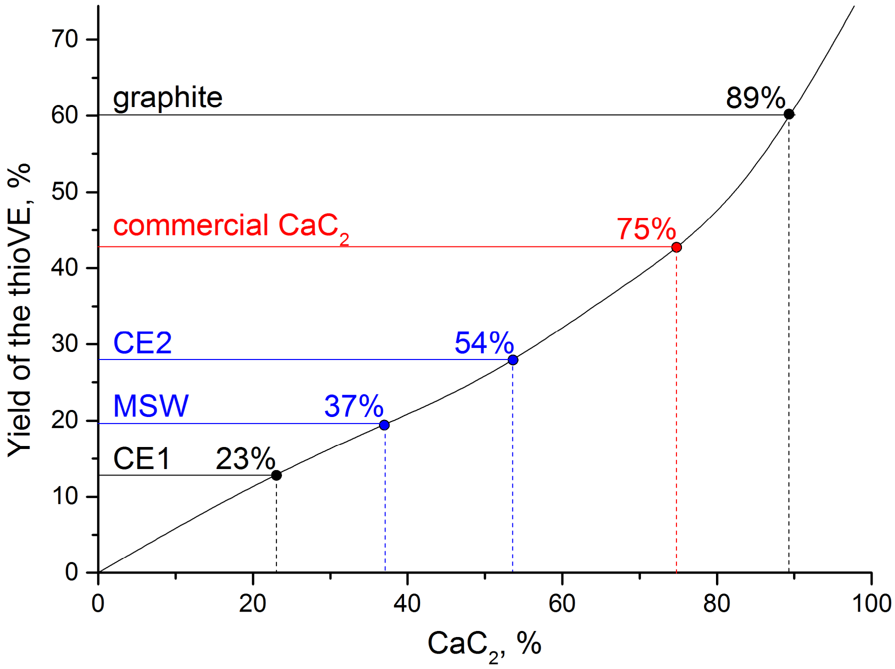

Figure S4. Determination of calcium carbide yields after the reaction in the oven.

Taking into account the excess calcium in the initial mixture, as well as the inorganic residue after combustion, for each sample, it is possible to calculate the carbide yield (based on carbon determined from pyrolysis data).

Table S4. Determination of the yield of calcium carbide obtained in an oven.

№	CM	C content (pyrolysis)	Inorg. residue (combustion)	C, mol	C, g	CM, g	Ca, mol*	Ca, g	Ca excess, g	Inorg. residue, g	M (CaC_2), g, theory	Max. CaC_2 content, mass%	CaC_2 content (NMR data), %	Yield (CaC_2), %
1	Graphite	100	0	0.12	1.50	1.50	0.069	2.75	0.257	0	4.0	94.0	89.3	95.0
2	CE1	45.4	4.95	0.03	0.37	0.82	0.017	0.69	0.064	0.041	1.0	90.5	23.1	25.5
3	CE2	87	0.75	0.03	0.37	0.43	0.017	0.69	0.064	0.003	1.0	93.7	53.6	57.2
4	MSW	38	9.5	0.03	0.37	0.99	0.017	0.69	0.064	0.094	1.0	86.4	37.2	43.1
5	MCC	15	2.3	0.03	0.37	2.50	0.017	0.69	0.064	0.057	1.0	89.2	0	0

*10 mol% calcium excess

$$m(Max\ CaC_2) = \frac{m(\text{theory } CaC_2)}{(m(\text{theory } CaC_2) + m(\text{excess } Ca) + m(\text{inorg. residue}))} \cdot 100\ \%$$

The yield of calcium carbide was calculated as the percentage of the carbide content in the mixture obtained from the spectrum to the maximum content of CaC_2 in the mixture.

5. NMR spectra.

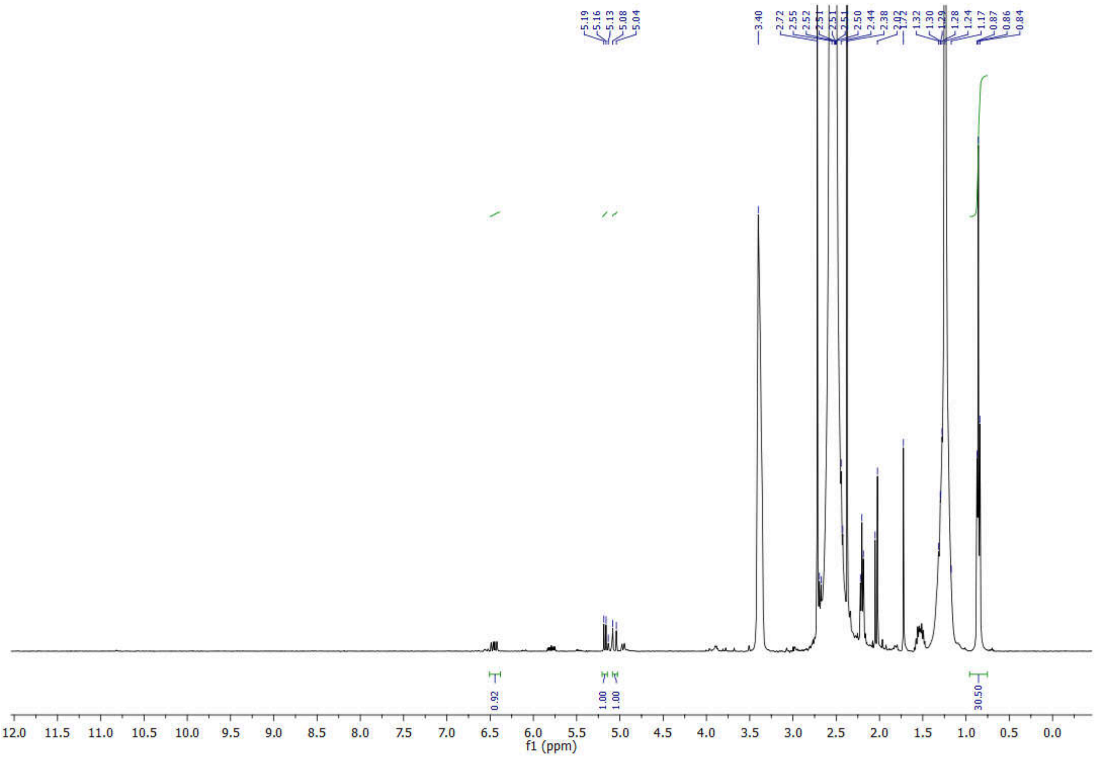

Figure S5. NMR ^{1}H spectra of the reaction mixture after the vinylation reaction (10 mg of calcium carbide).

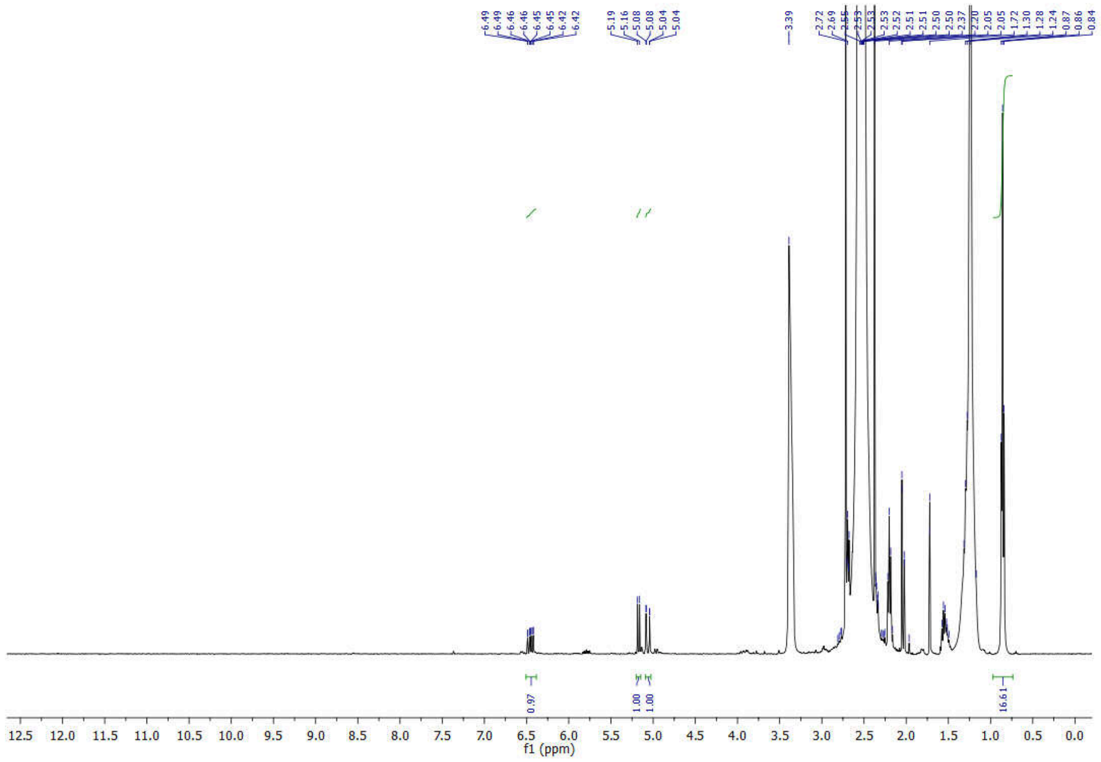

Figure S6. NMR ^{1}H spectra of the reaction mixture after the vinylation reaction (20 mg of calcium carbide).

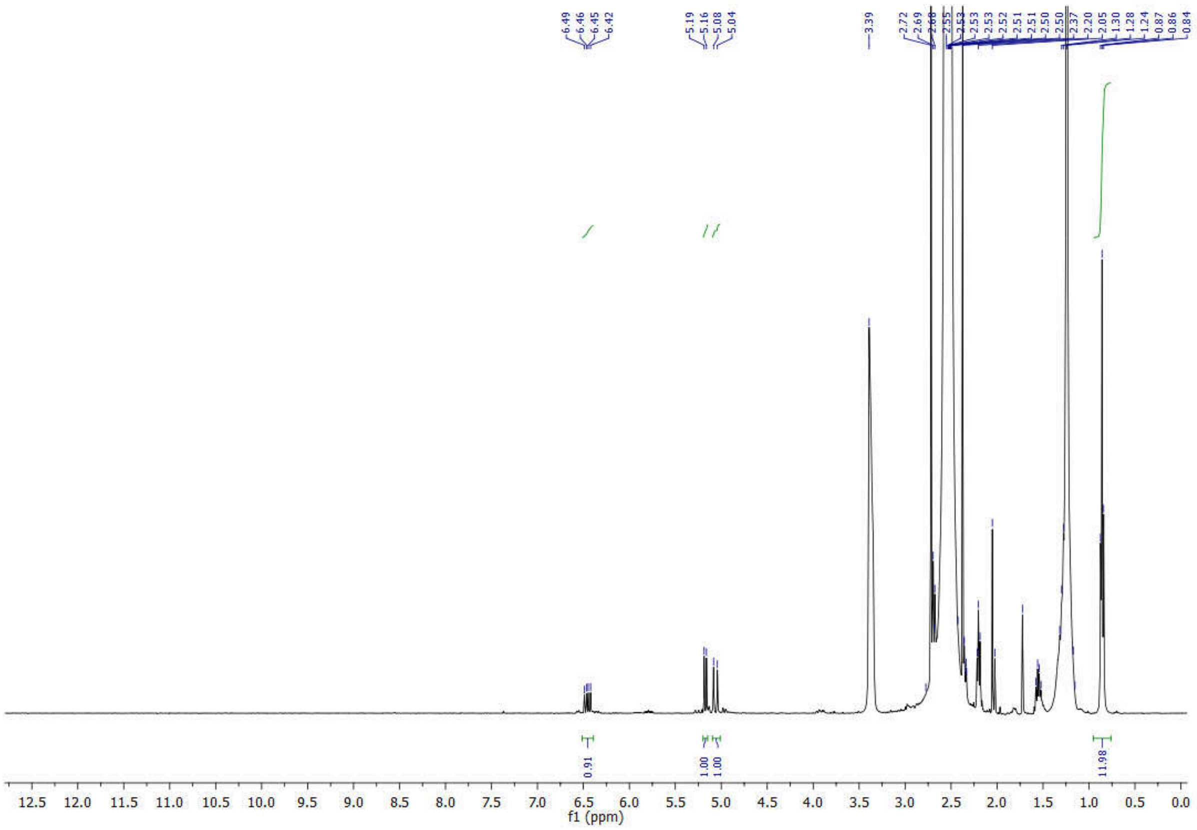

Figure S7. NMR ^1H spectra of the reaction mixture after the vinylation reaction (30 mg of calcium carbide).

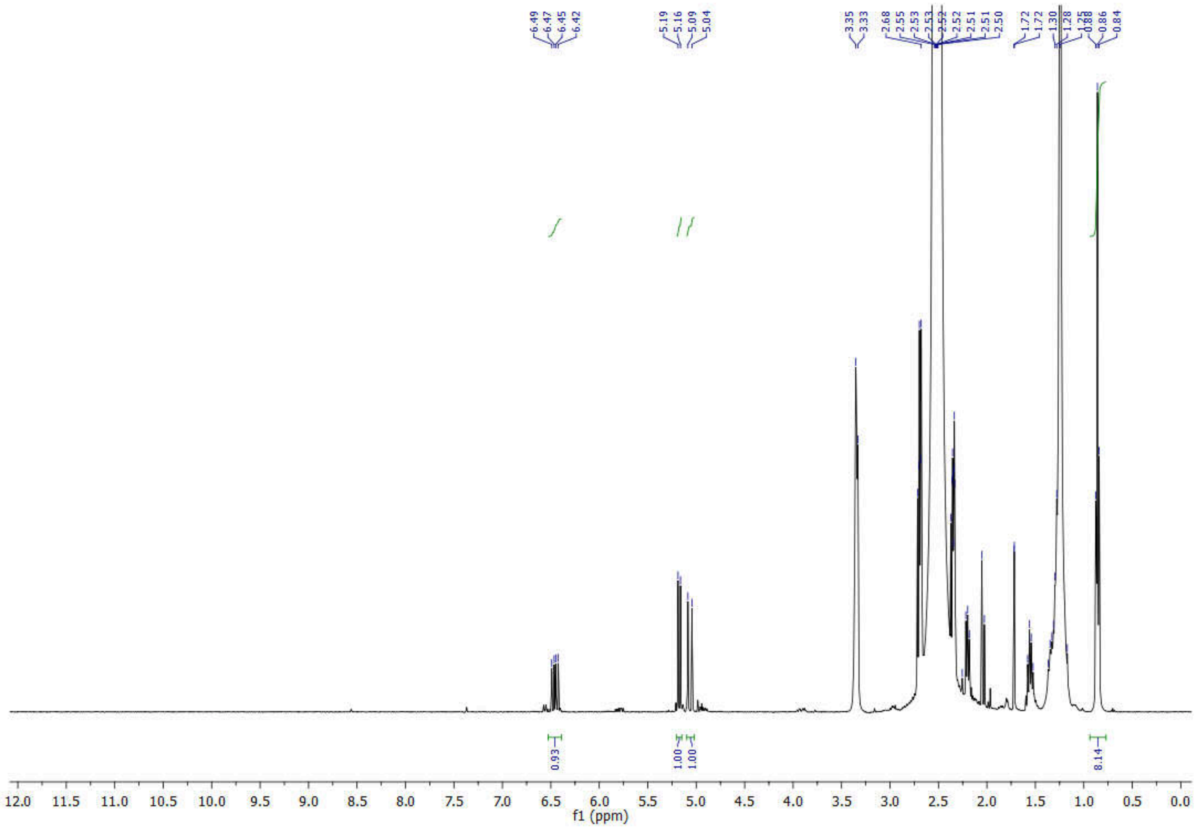

Figure S8. NMR ^1H spectra of the reaction mixture after the vinylation reaction (40 mg of calcium carbide).

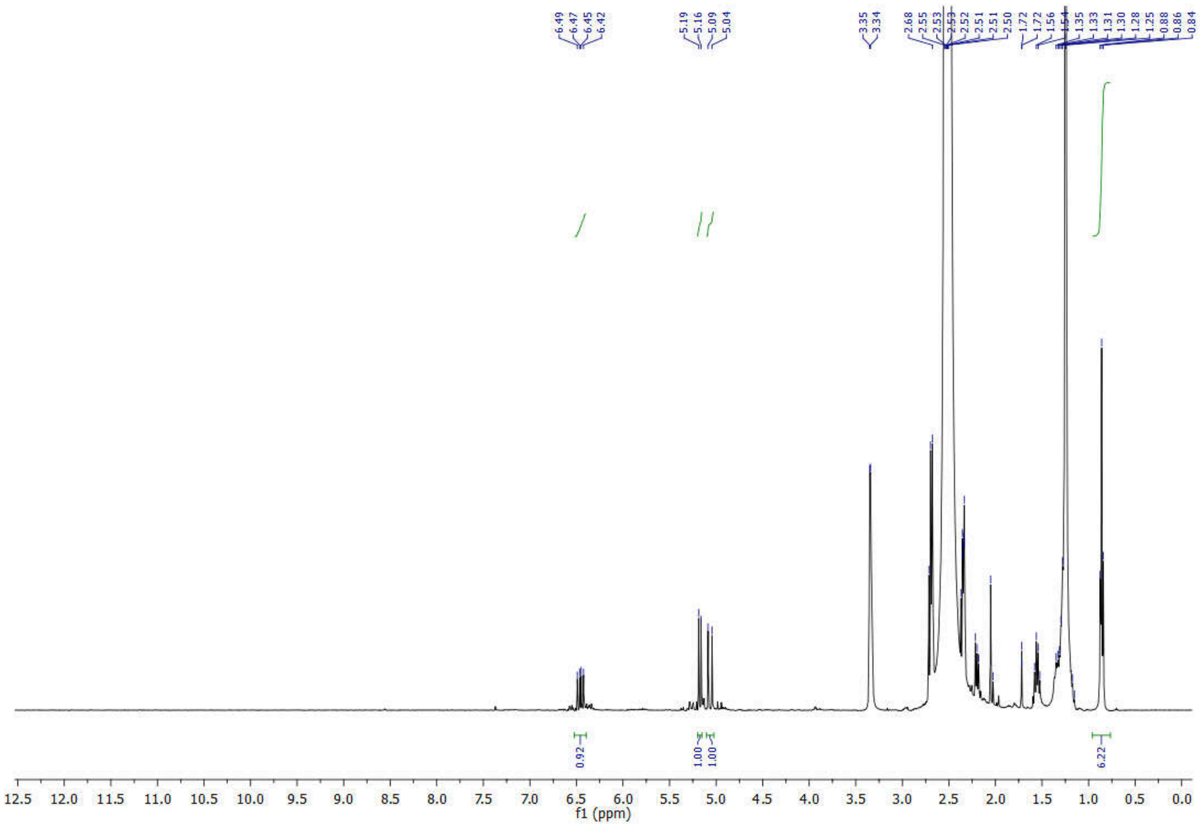

Figure S9. NMR ^{1}H spectra of the reaction mixture after the vinylation reaction (50 mg of calcium carbide).

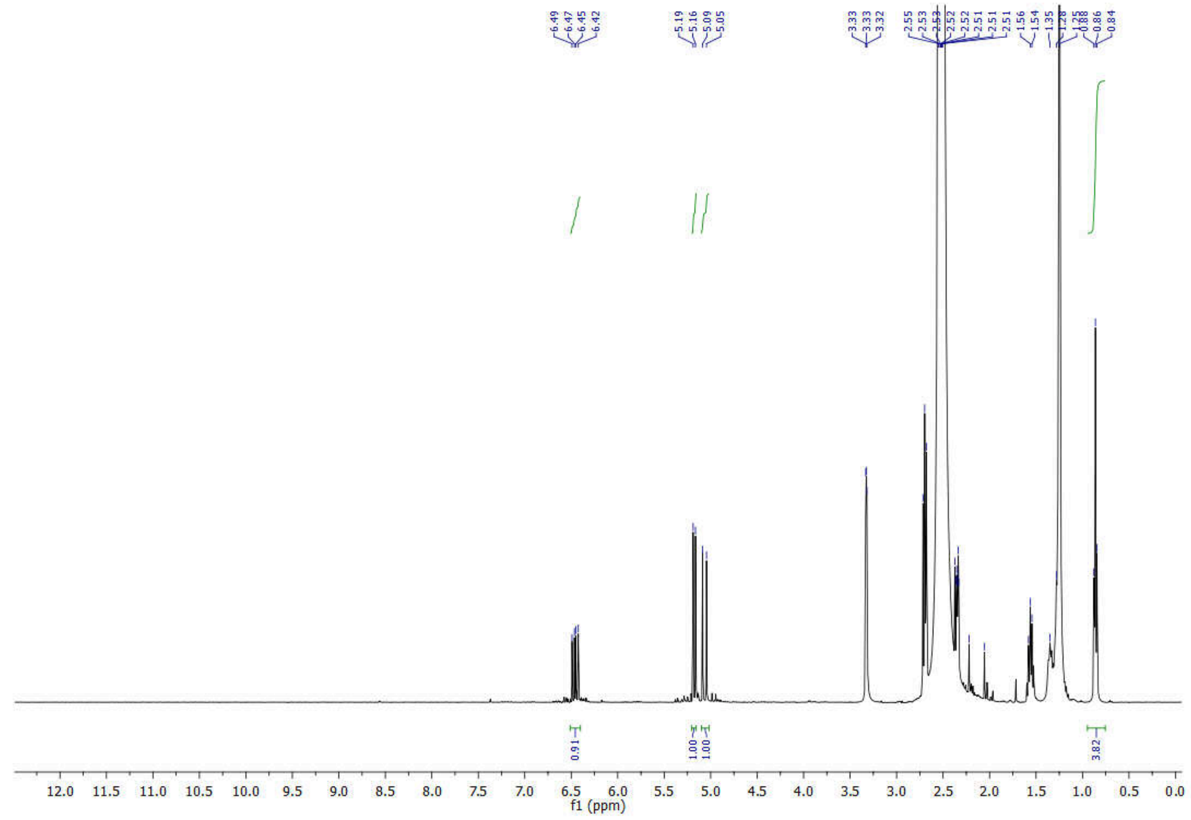

Figure S10. NMR ^{1}H spectra of the reaction mixture after the vinylation reaction (60 mg of calcium carbide).

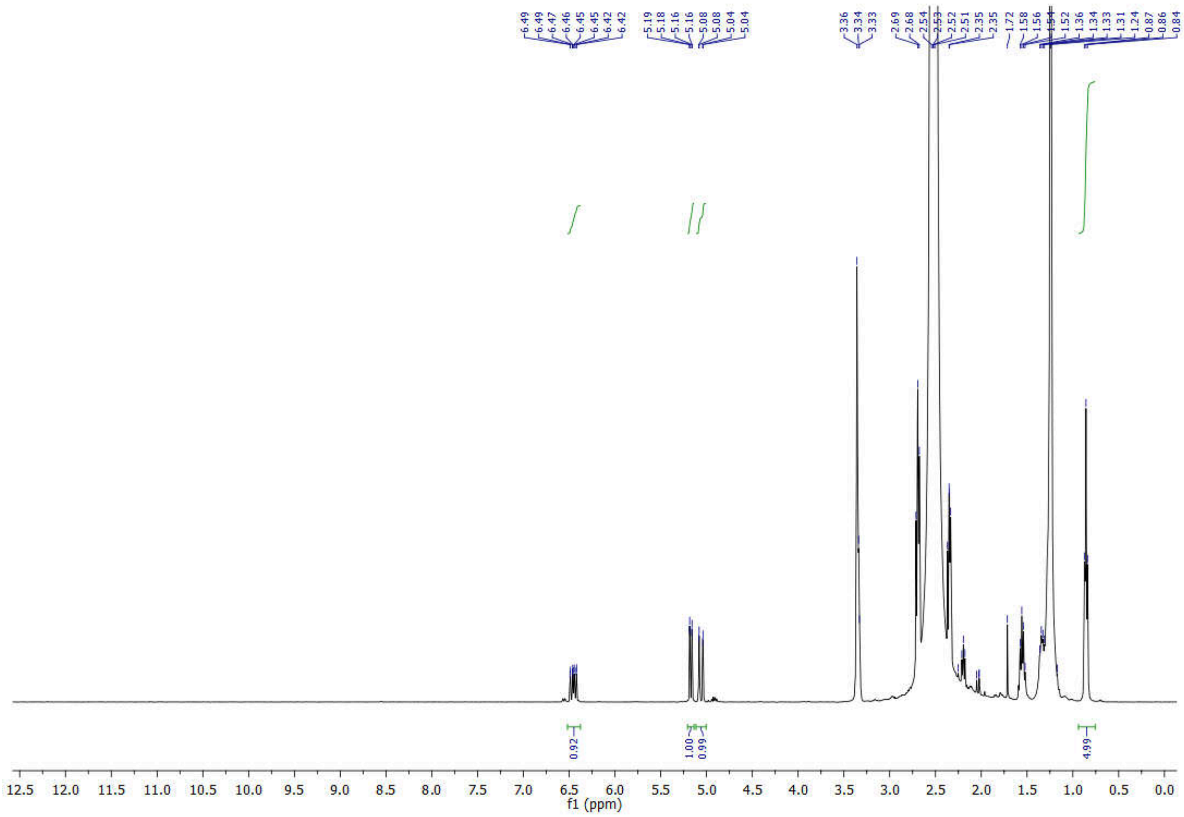

Figure S11. NMR ^{1}H spectra of the reaction mixture after the vinylation reaction of calcium carbide derived from graphite.

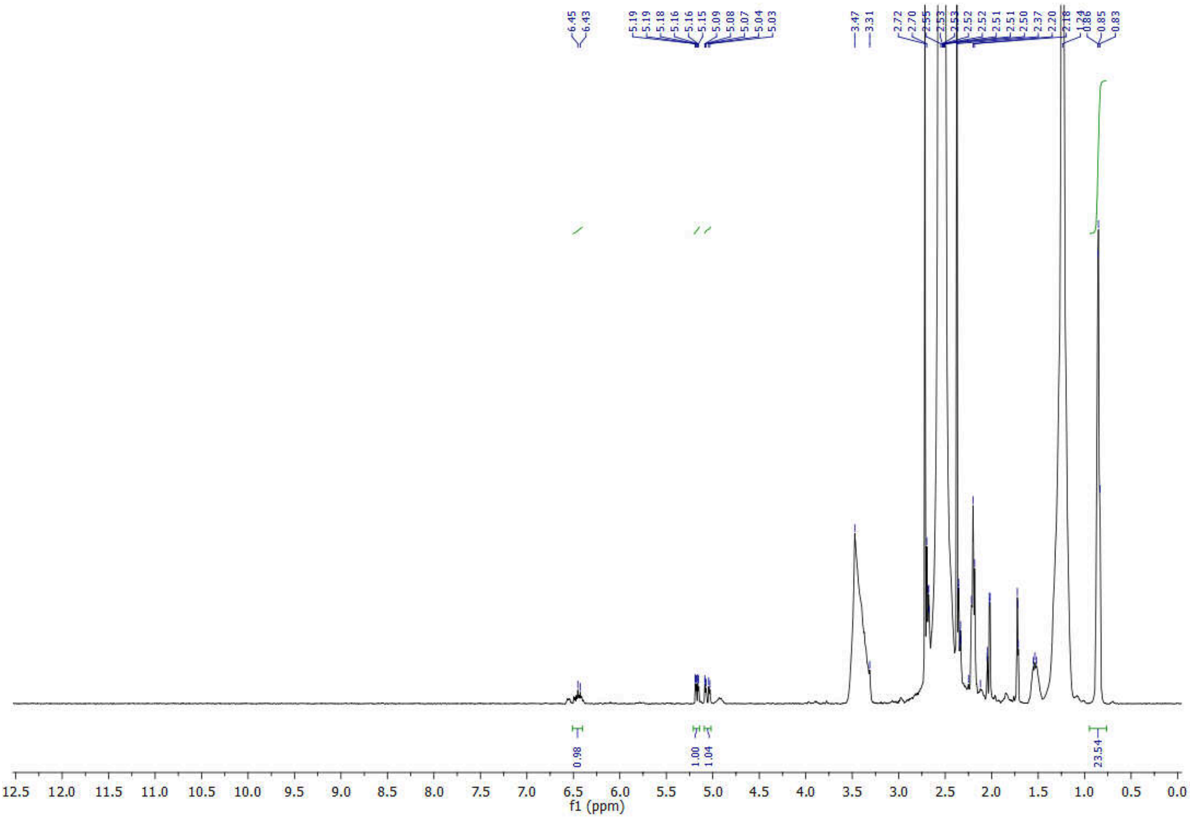

Figure S12. NMR ^{1}H spectra of the reaction mixture after the vinylation reaction of calcium carbide derived from CE1.

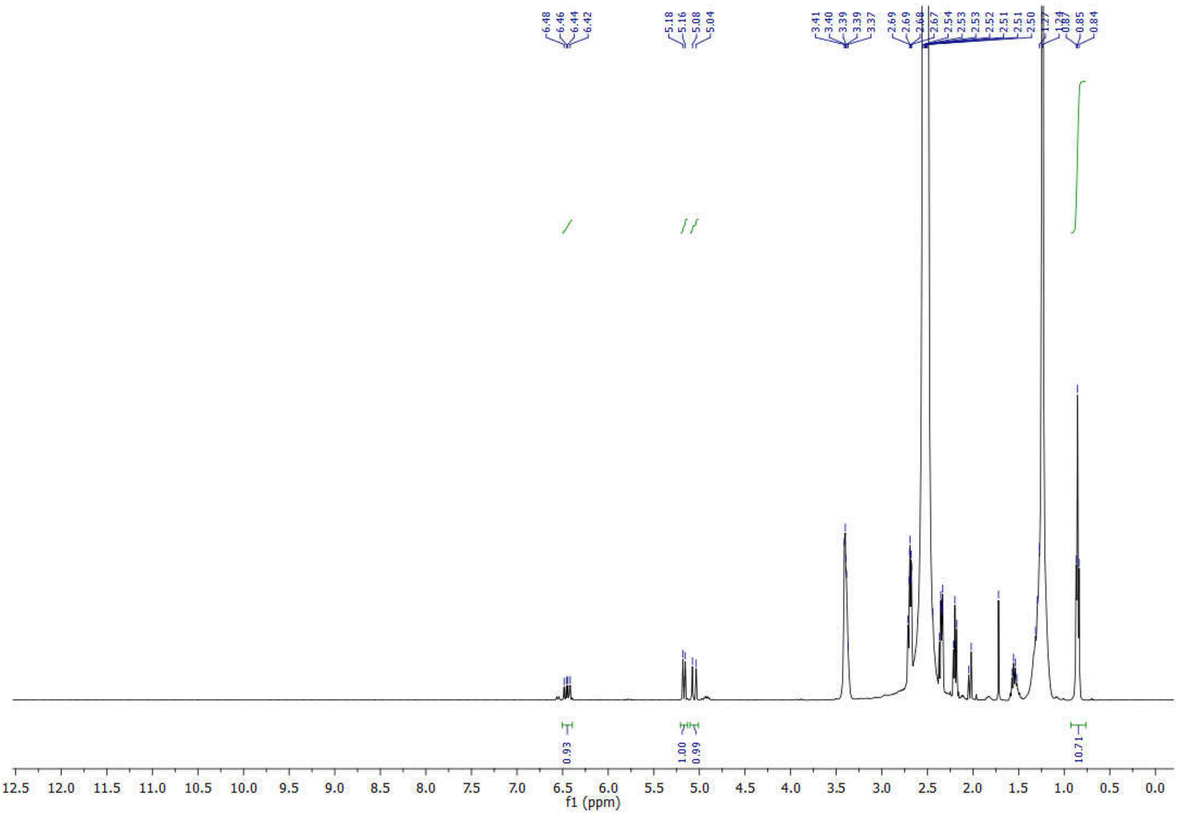

Figure S13. NMR ^{1}H spectra of the reaction mixture after the vinylation reaction of calcium carbide derived from CE2.

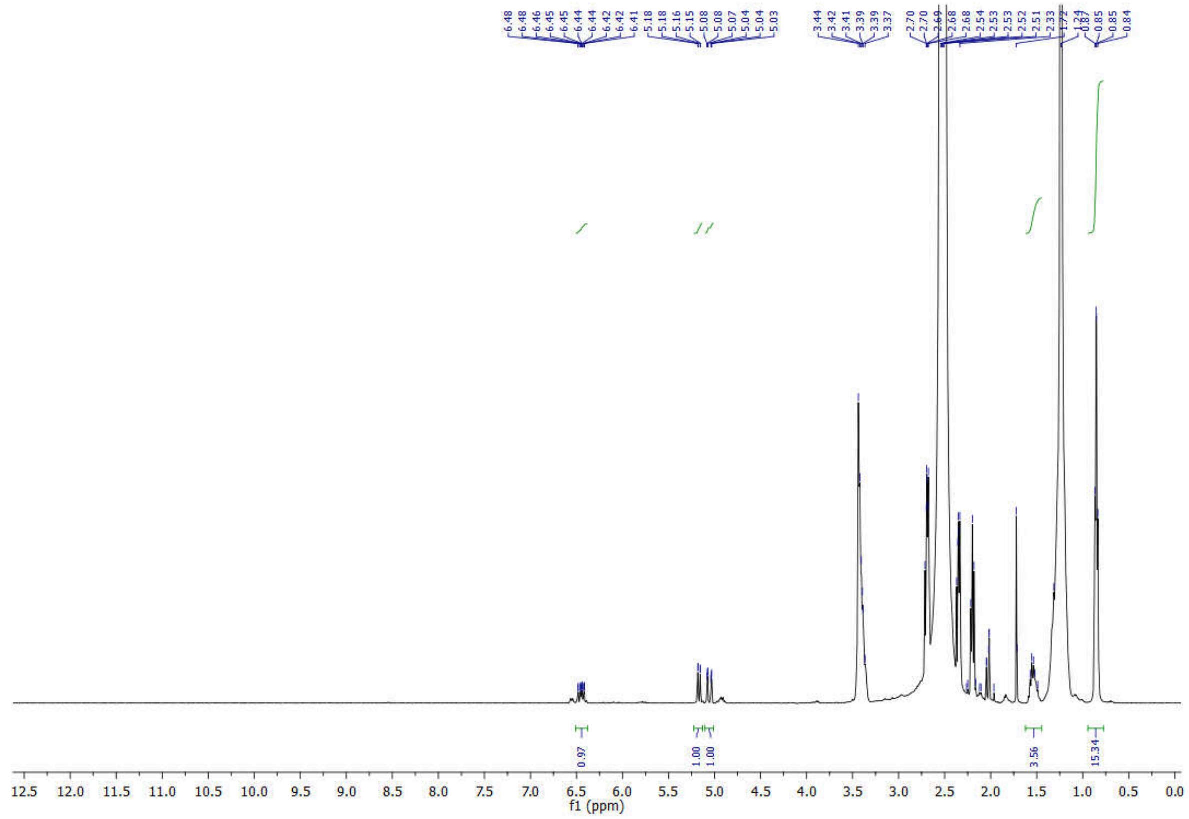

Figure S14. NMR ^{1}H spectra of the reaction mixture after the vinylation reaction of calcium carbide derived from MSW.

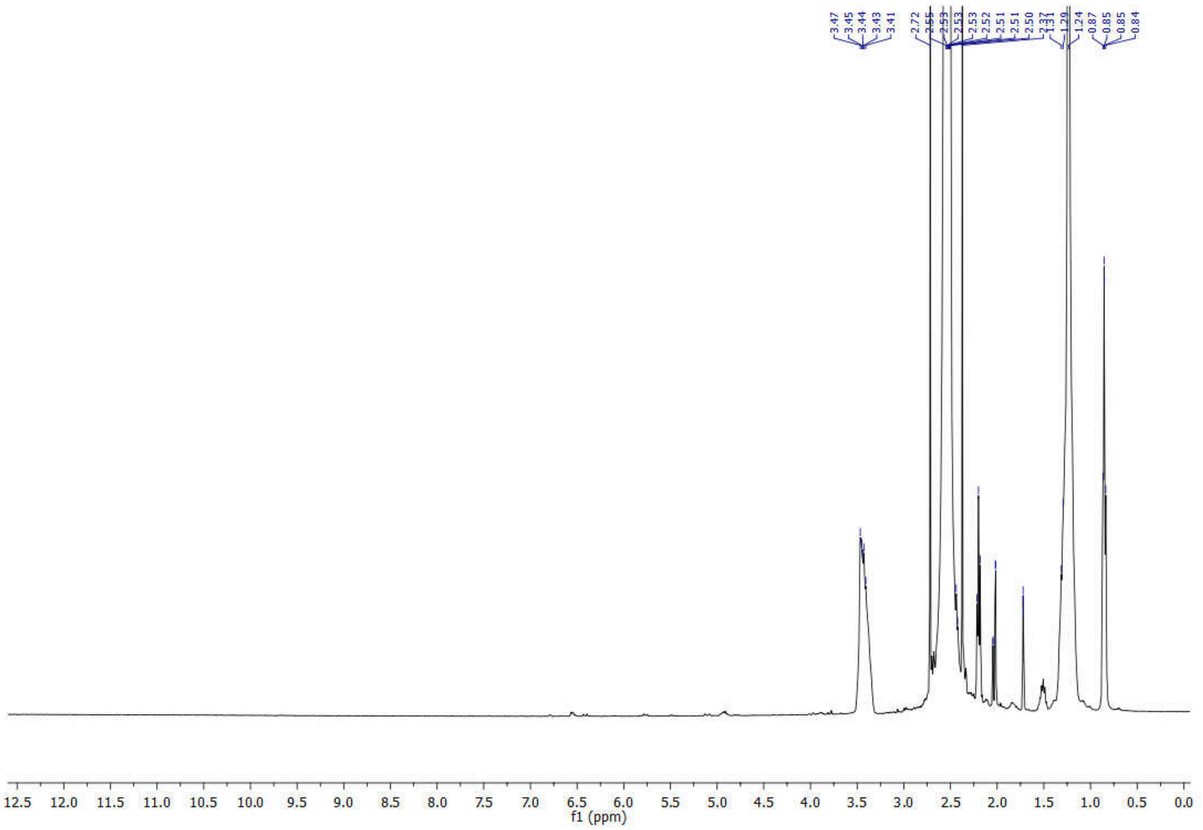

Figure S15. NMR ^{1}H spectra of the reaction mixture after the vinylation reaction of calcium carbide derived from MCC.

References

[1] K. S. Rodygin, V. P. Ananikov, *Green Chem.* **2016**, *18*, 482-486.
